# Long-term visual function and refractive changes after vitrectomy for stage 4 retinopathy of prematurity

**DOI:** 10.1007/s00417-025-06801-0

**Published:** 2025-03-22

**Authors:** Kuniko Tachibana, Chiharu Iwahashi, Kazuki Kuniyoshi, Shunji Kusaka

**Affiliations:** Department of Ophthalmology, Faculty of Medicine, Kindai University, Osakasayama City, 377-2 Ohnohigashi, Osaka, 589-8511 Japan

**Keywords:** Refractive errors, Retinopathy of prematurity, Retrospective studies, Visual acuity, Vitrectomy, Lens-sparing vitrectomy

## Abstract

**Purpose:**

This study aimed to investigate longitudinal changes in best-corrected visual acuity (BCVA) and refraction in patients following vitrectomy for stage 4 retinopathy of prematurity (ROP).

**Methods:**

We conducted a retrospective review of 42 eyes from 25 patients (35 eyes with stage 4A, 7 eyes with stage 4B) who had successful vitrectomy for stage 4 ROP and were followed for at least 8 years. Postoperative BCVAs and refractive errors between ages 5 and 8 years were compared. Factors related to BCVA at ages 5 and 8, as well as their differences, were analyzed.

**Results:**

In stage 4A ROP eyes, the mean logMAR BCVA improved significantly from 0.83 (20/135) to 0.63 (20/85) (*p* < 0.001) and a myopic shift of 1 D or more occurred in 21 eyes (61.8%) between ages 5 and 8. In the poor BCVA group at age 5 in the stage 4A eyes, dominant eyes showed a trend of BCVA improvement by ages 5–8 (*p* = 0.06). Multiple regression analysis of the patients with stage 4A ROP showed that phakic and dominant eyes at age 5 were independently associated with better BCVA at ages 5 and 8 (*p* = 0.006 and 0.016 for age 5; *p* = 0.009 and 0.002 for age 8). No significant BCVA improvement was noted in stage 4B ROP eyes during the same period.

**Conclusion:**

This study indicated the possibility of continued visual improvement beyond age 5 in patients who underwent vitrectomy for stage 4A ROP, although a myopic shift occurred concurrently.

**Key messages:**

***What is known***

- Previous studies have reported long-term visual prognosis and refractive errors at specific time points after vitrectomy for ROP in small case series, but there has been limited research on serial changes.

***What is new***

- This study demonstrates the potential for ongoing visual improvement beyond age 5 and highlights longitudinal myopic changes between ages 5 and 8 in patients who underwent vitrectomy for stage 4A ROP.
- In patients with poor visual acuity at age 5, being the dominant eye was significantly associated with improved visual acuity by age 8.
- The findings indicate that postoperative visual acuity in ROP patients gradually improves, even as myopia progresses, underscoring the need for long-term follow-up.

**Supplementary Information:**

The online version contains supplementary material available at 10.1007/s00417-025-06801-0.

## Introduction

Retinopathy of prematurity (ROP) is a leading cause of childhood blindness globally [1, 2]. Laser photocoagulation of the avascular retina and intravitreal injection of anti-vascular endothelial growth factor (VEGF) are currently the mainstay of treatment for ROP [3, 4]. However, despite timely and intensive treatment, some patients may still experience traction retinal detachment and require surgical intervention [5]. The prevailing opinion is that vitrectomy should be performed at stage 4A (partial retinal detachment not involving the macula), as the timing has been associated with better surgical outcomes compared to stage 4B (partial retinal detachment involving the macula) or stage 5 (total retinal detachment) [6, 7].

While the surgical outcomes of vitrectomy for stage 4 ROP are relatively well established [8–10], information regarding their long-term results, particularly functional outcomes, is limited [11–13]. Based on our clinical observations, many patients experience gradual improvements in visual acuity even after age 4 or 5, when developing children typically reach adult-level visual acuity [14, 15]. This study aimed to investigate whether the visual function of patients who underwent vitrectomy for stage 4 ROP continues to change after age 5. Additionally, we analyzed factors that may influence long-term visual acuity improvement and examined changes in refraction during this period.

## Materials and methods

### Participants

We reviewed the medical records of patients who underwent successful vitrectomy for stage 4A (4A group) or stage 4B (4B group) ROP, with follow-up data collected between ages 5 and 8. Vitrectomy was performed at Osaka University Hospital or Kindai University Hospital in Osaka, Japan, between June 2006 and May 2014. All patients underwent either lens-sparing vitrectomy (LSV) or vitrectomy with lensectomy, as previously described [16]. All surgeries were conducted by a single surgeon (S.K.). Following surgery, patients were monitored at Kindai University Hospital. All patients were referred from other hospitals where they received laser ablation as the initial treatment for ROP. Only patients who could undergo best-corrected visual acuity (BCVA) testing at ages 5 and 8 years were included. Those whose BCVAs could not be assessed using Landolt ring tests at age 5, who did not have examinations at age 8 or who underwent additional intraocular surgery before age 8 were excluded from the study.

### Measurement of the BCVA and refractive errors

BCVA was assessed monocularly using the Landolt ring test at a distance of 5 m annually from ages 5 to 8. For statistical analysis, decimal visual acuities were converted to logMAR values. In this study, a BCVA of 20/60 was considered visual impairment (> 0.52, logMAR acuity), in line with World Health Organization definitions [17]. BCVA improvement or deterioration was defined as a change in logMAR BCVA of 0.2 or more [18]. The dominant eye was identified as the one with a BCVA of 0.2 logMAR or better than the fellow eye, based on prior reports [19]. Refractive errors were evaluated based on subjective BCVA correction, as few children could undergo autorefraction. A change in spherical equivalent (SE) of 1.0 D or more was classified as a myopic or hyperopic shift.

### Statistical analysis

For statistical summaries, continuous variables were reported as medians with interquartile ranges (IQR) or means with standard deviations, as appropriate. Categorical variables were presented as frequencies and percentages (%). The Mann–Whitney U test or Fisher’s exact test was used to compare categorical variables, depending on the situation. Multiple regression analysis was conducted for the 4A group to examine factors associated with BCVA at ages 5 and 8. This analysis was performed in three ways, using each BCVA measurement as the outcome variable. Eight factors were selected as potential explanatory variables: gestational age (GA), birth weight, sex, number of vitrectomies, postconceptional age (PCA) at the time of primary vitrectomy, history of anti-VEGF injection, phakic eye and dominant eye at age 5. For eyes with visual acuity of hand motion, the logMAR equivalent was recorded as 2.30 [20]. Optimal variables were chosen using a stepwise method. To assess the significance of serial age-related changes in BCVA and SE from ages 5–8, the Friedman test (two-way analysis of variance by ranks) was used. Mean imputation was applied for missing values to enhance the robustness of the results. Additionally, as a secondary analysis, Spearman’s rank correlation coefficient was calculated for phakic eyes in the 4A group to evaluate associations among the explanatory factors for BCVA and SE at ages 5 and 8, as well as the differences between these ages. All statistical analyses were conducted using StatFlex version 7.0 (Artech Co. Ltd., Osaka, Japan).

## Results

### Patient demographics

**Fig. 1:**
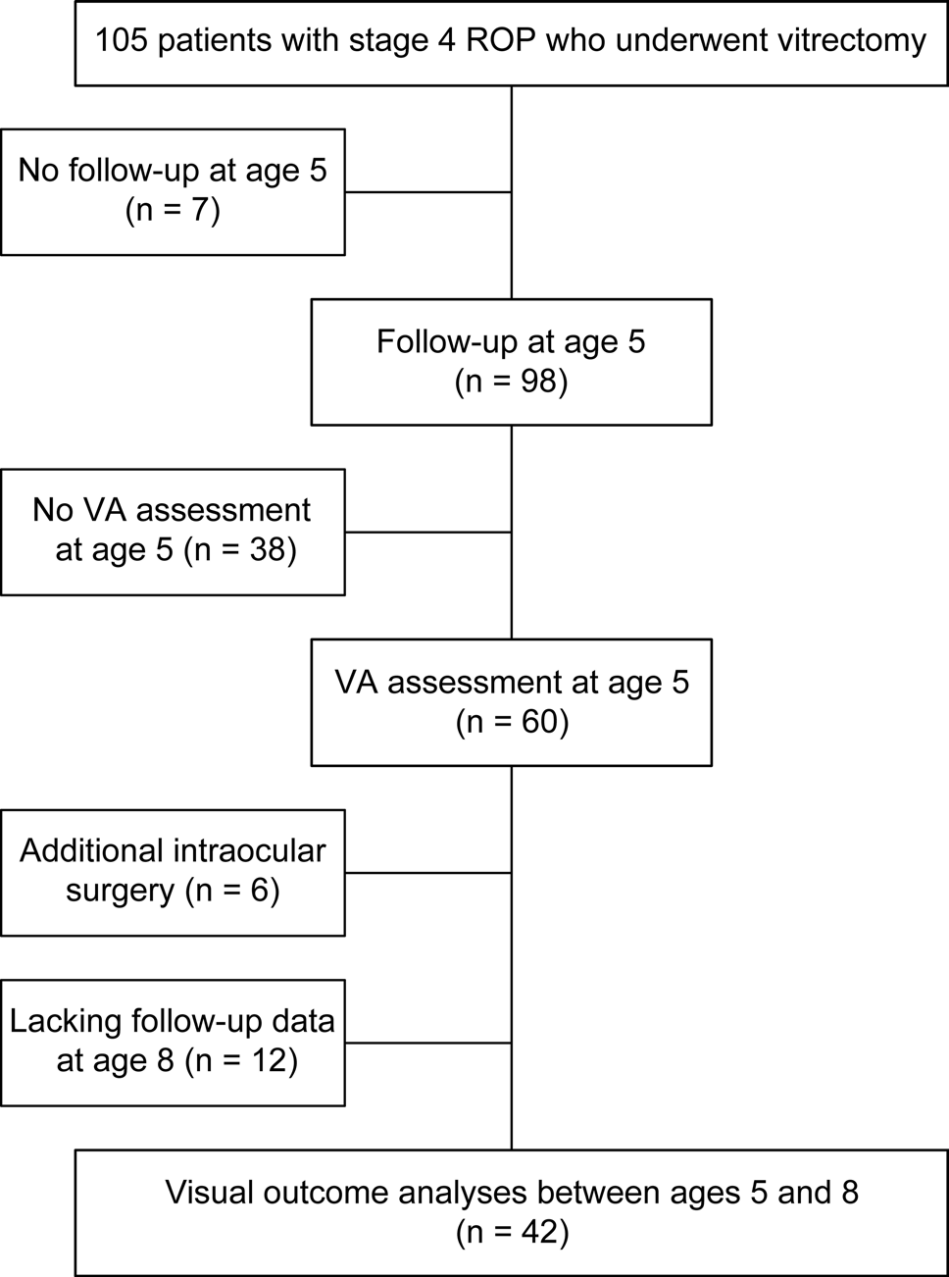
Study schematic. ROP, retinopathy of prematurity; VA, visual acuity

During the study period, 105 eyes from 69 patients with stage 4 ROP underwent vitrectomy. Of these, 35 eyes from 25 patients in the 4A group and 7 eyes from 6 patients in the 4B group met the inclusion criteria (details shown in Fig. 1). The demographics of the patients are presented in Table 1. Of the included eyes, 31 underwent LSV and 11 underwent vitrectomy with lensectomy.

**Table 1:** Clinical features of eyes with stage 4 retinopathy of prematurity

		stage 4A (35 eyes of 25 patients)	stage 4B (7 eyes of 6 patients)
Sex	Number of patients (%)		
Girl		14 (56.0)	3 (50.0)
Boy		11 (44.0)	3 (50.0)
GA (day)	Median	175.0	180.0
	Range	154—190	161—238
BW (g)	Median	640.0	710.5
	Range	459—962	514—1,652
PCA at primary vitrectomy (days)	Median	289.5	337
	Range	236 – 465	254—410
Number of vitrectomy	Mean	1.1	1.4
	Range	1–2	1–2
Intravitreal bevacizumab injection	Number of eyes (%)		
Yes		7 (20.0)	1 (14.2)
No		28 (80.0)	6 (85.7)
PO lens status	Number of eyes (%)		
Phakia		26 (74.3)	5 (71.4)
Aphakia		9 (25.7)	2 (28.6)

*GA* Gestational age (day), *BW* Birth weight (g), *PCA* post-conceptional age, *PO* post-operative

### Serial changes in BCVA

**Fig. 2:**
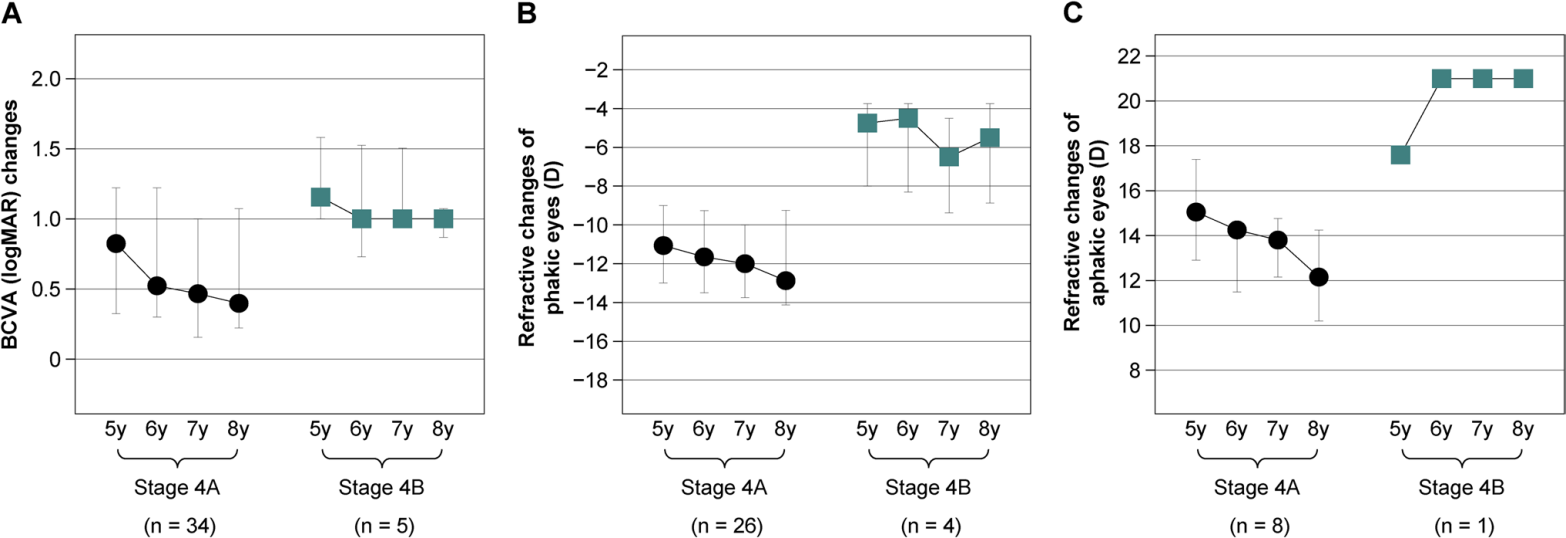
Visual acuity and refractive changes following vitrectomy for stage 4 retinopathy of prematurity. **A**: Serial BCVA changes for eyes in the 4A and 4B groups between the ages of 5 and 8 years. **B**: Serial refractive changes in phakic eyes from the 4A and 4B groups between the ages of 5 and 8 years. **C**: Serial refractive changes in aphakic eyes from the 4A and 4B groups between the ages of 5 and 8 years. Black circles or squares indicate the median values, while the lines represent the interquartile ranges. BCVA, best-corrected visual acuity; D, diopter

Figure 2A illustrates the changes in BCVA for patients in the 4A group between ages 5 and 8. Compared to their BCVA at age 5, 16 eyes from 12 patients (45.7%) in the 4A group showed improvement at age 8, while BCVA remained stable in 18 eyes from 14 patients (51.4%) and worsened in 1 eye (2.9%). The median logMAR BCVA (IQR; range) was 0.82 (20/132) (0.90; − 0.08–1.70) at age 5 and improved to 0.40 (20/50) (0.85; − 0.08–2.00) at age 8 (*p* < 0.001).

In the 4B group, BCVA improved in 3 eyes from 3 patients (43%) at age 8, while it remained unchanged in 4 eyes from 3 patients (57%) compared to age 5. The median logMAR BCVA (IQR; range) was 1.15 (20/283) (0.58; 0.40–1.70) at age 5 and 1.00 (20/200) (0.21; 0.05–1.70) at age 8 (*p* = 0.128).

Overall, significant improvement in BCVA was observed in the 4A group between ages 5 and 8, while the improvement in the 4B group was not significant.

### Serial changes in refraction

Figure 2B depicts the changes in refraction between ages 5 and 8, stratified by phakic/aphakic status. In the 4A group, refraction testing was not performed on 1 eye at age 8. Among the 34 eyes in this group that had measurable refraction, myopic shifts were seen in 21 eyes (61.8%) and hyperopic shifts in 6 eyes (17.6%) between ages 5 and 8. For the 26 phakic eyes in the 4A group, the mean SEs were − 10.9 ± 3.3 D at age 5 and − 11.9 ± 3.5 D at age 8. In the eight aphakic eyes in the 4A group, the mean SEs were + 15.0 ± 3.1 D at age 5 and + 12.4 ± 3.1 D at age 8.

In the 4B group, refraction testing was not conducted for 2 eyes of 2 patients at age 5. Among the 5 eyes in the 4B group with measurable refraction, 1 eye (20%) exhibited a myopic shift, and 1 eye (20%) exhibited a hyperopic shift between ages 5 and 8. In the 4 phakic eyes of the 4B group, the mean SEs were − 5.9 ± 3.5D at age 5 and − 6.3 ± 3.9 D at age 8.

### Subanalysis by good and poor BCVA groups at age 5

**Fig. 3:**
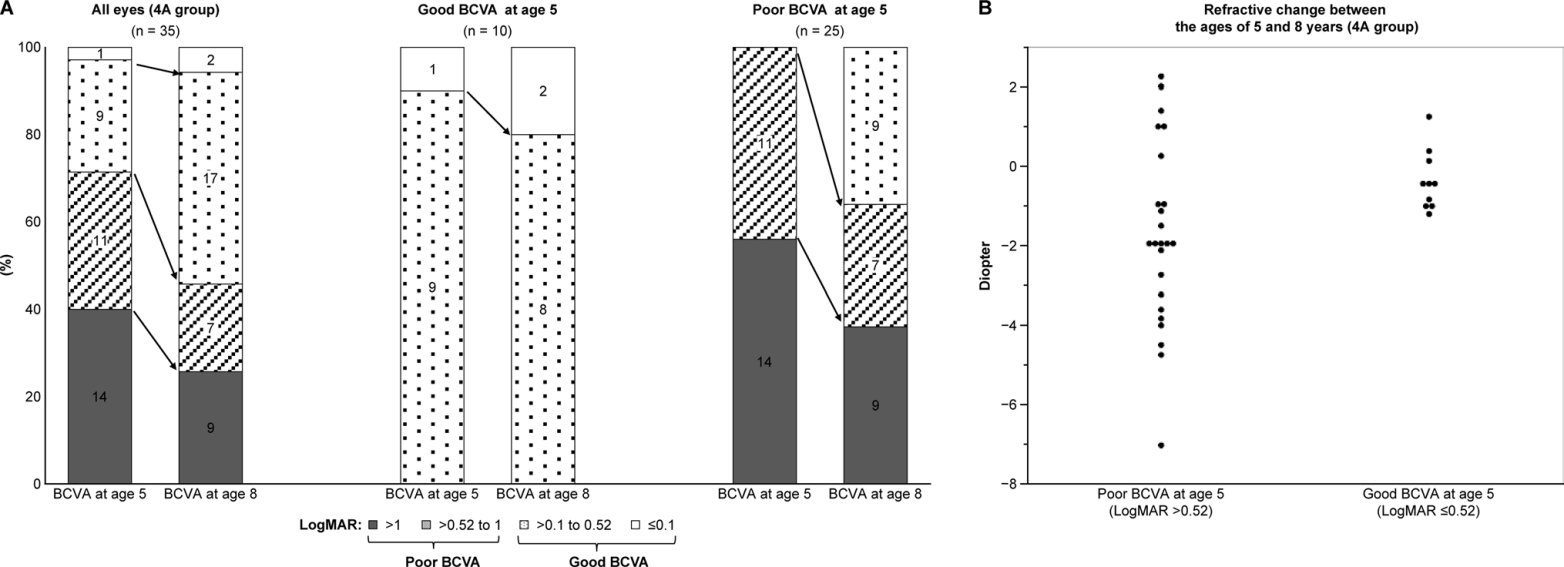
Relationships between BCVA and refractive error at ages 5 and 8. **A**: Bar graph illustrating the relationship between good and poor BCVA at age 5 and BCVA at age 8. **B**: Comparison of refractive changes from ages 5 to 8 based on poor and good BCVA at age 5. BCVA, best-corrected visual acuity

The 35 eyes in the 4A group were divided into two categories based on their BCVA at age 5: the good BCVA group (logMAR ≤ 0.52) (≥ 20/60) and the poor BCVA group (logMAR > 0.52) (< 20/60). We then assessed changes in BCVA and myopic shifts between ages 5 and 8. The results indicated that the BCVAs of all 10 eyes in the good BCVA group at age 5 remained in the same category at age 8. In contrast, among the 25 eyes in the poor BCVA group at age 5, 9 eyes (36%) showed improvement and were categorized as good BCVA at age 8, while the BCVAs of the remaining 16 eyes stayed the same (Fig. 3A). We also analyzed which eyes in the poor BCVA group at age 5 were more likely to demonstrate improvement. In this group, the mean change in logMAR BCVA from ages 5 to 8 for the dominant eyes at age 5 was 0.41 ± 0.14 (20/51 ± 20/28), suggesting a trend toward greater improvement compared to the change of 0.21 ± 0.25 (20/32 ± 20/36) in the nondominant eyes at age 5 (*p* = 0.060). This indicates that the dominant eye at age 5 tended to be associated with improved BCVA. Next, we analyzed the 25 eyes from the poor BCVA group at age 5 to assess the relationship between dominant and nondominant eyes at that age and their vision status at age 8. In this analysis, 5 out of 6 dominant eyes (83.3%) achieved good vision by age 8, compared to only 4 out of 19 nondominant eyes (21.1%) (*p* = 0.006). This suggests that even with poor vision at age 5, dominant eyes have a significantly higher likelihood of achieving good vision by age 8. Additionally, excluding 1 eye for which refractive values could not be measured, we examined the relationship between BCVA at age 5 and myopic shift. The mean refractive change between ages 5 and 8 was − 1.78 ± 2.31D in the poor BCVA group at age 5, which was significantly greater than the change in the good BCVA group at age 5 (− 0.37 ± 0.76D) (*p* = 0.027) (Fig. 3B).

### Analyses of factors associated with BCVA at ages 5 and 8

Regression analysis was conducted on data from patients in the 4A group to identify potential predictors of BCVA at ages 5 and 8, as well as predictors for the change in BCVA between these ages.

The analysis revealed that the status of the phakic status and whether the eye was dominant at age 5 were independent predictors of good BCVA at both ages 5 and 8 (*p* = 0.006 and 0.016 for age 5; *p* = 0.009 and 0.002 for age 8), as indicated in Table 2. This conclusion was supported by the scattergrams of BCVA categorized by the phakic and dominant eye statuses in Supplementary Figure. S1. However, no factors were identified that could predict the difference in BCVA between the two ages.

**Table 2:** Multiple regression analysis of best corrected visual acuity at 5 and 8 years for stage 4A (*n* = 35)

**BCVA at age of 5 years old; R = 0.5509**					
	β	SE (β)	stdβ	*t*-value	*P*-value
Lens	−0.5316	0.1786	−0.4415	−2.9766	0.00551
Dominant eye at age 5	−0.4552	0.1786	−0.3779	−2.5483	0.01582
**BCVA at age of 8 years old; R = 0.5992**					
	β	SE (β)	stdβ	*t*-value	*P*-value
Lens	−0.4714	0.1705	−0.3934	−2.7652	0.00936
Dominant eye at age 5	−0.5920	0.1705	−0.4941	−3.4724	0.00150
**Difference of BCVA between 5 and 8 years old; R = 0.2996**					
	β	SE (β)	stdβ	*t*-value	*P*-value
Lens	0.06024	0.08775	0.1164	0.6865	0.49733
Dominant eye at age 5	−0.1368	0.08775	−0.2643	−1.5591	0.12881

*BCVA* best corrected visual acuity (logMAR acuity), Lens; phakia converted 1, aphakia converted 0, Dominant eye; dominant eye converted 1, non-dominant converted 0

*R* multiple correlation coefficient, *SE* standard error

In the second analysis using the Spearman correlation coefficient, focusing on phakic eyes, the only significant factor associated with logMAR BCVA at both ages 5 and 8 was GA, which showed a strong correlation with BCVA at both ages (rS =  − 0.481, *p* = 0.013; rS =  − 0.489, *p* = 0.011), as illustrated in Supplementary Figure. S2. This suggests that a shorter GA is associated with poorer BCVA. However, no factors were identified as correlated with refractive changes between ages 5 and 8. Additionally, these analyses were not conducted for the stage 4B group due to the small sample size.

## Discussion

This study examined the long-term visual outcomes and refractive changes in patients who underwent vitrectomy for stage 4 ROP. Among patients with stage 4A ROP, the median logMAR BCVA was 0.82 (20/132) at age 5 and improved to 0.40 (20/50) at age 8 (*p* < 0.001). A myopic shift of 1 D or more was noted in 21 eyes (61.8%) between ages 5 and 8. In the group with poor BCVA at age 5, being the dominant eye was associated with improvements in BCVA from ages 5 to 8. Moreover, multiple regression analysis indicated that both the phakic eye status and being the dominant eye at age 5 were independently associated with better BCVA at both ages 5 and 8.

Several studies have examined visual prognosis after vitrectomy for advanced ROP. Özsaygili et al. analyzed the visual outcomes of 121 eyes from 82 infants with stage 4A/4B ROP who underwent LSV or vitrectomy with lensectomy, finding a mean logMAR BCVA of 1.12 ± 0.34 (20/264 ± 20/44) for stage 4A and 1.34 ± 0.32 (20/438 ± 20/42) for stage 4B at the 3-year follow-up [9]. Singh et al. conducted a study with a longer observation period, reporting a mean logMAR of 0.92 (20/166) for stage 4A and 1.63 (20/853) for stage 4B in eyes following LSV with a minimum follow-up of 5 years [11]. Karacorlu et al. noted a mean postoperative Snellen visual acuity of 20/550 for stage 4A and 20/1,600 for stage 4B, with an average follow-up of 6.9 years [12]. In this study, the median logMAR BCVAs at age 5 were 0.82 (20/132) for the 4A group and 1.15 (20/283) for the 4B group, while at age 8, they were 0.40 (20/50) and 1.00 (20/200), respectively. These values appear to be slightly better than those reported in previous studies. This improvement may be due to careful management practices, including regular prescriptions for glasses or contact lenses based on refractive errors. Moreover, advancements in vitreous surgery, such as the use of smaller gauge instruments that reduces surgical invasiveness, along with the introduction of anti-VEGF drugs, may have contributed to the improved visual outcomes. Preoperative anti-VEGF therapy for eyes with high vascular activity might have enabled earlier surgery and could be linked to intra- or post-operative complications, such as vitreous hemorrhage and/or reproliferation. However, the effectiveness of preoperative anti-VEGF therapy needs to be explored in future studies. In terms of surgical stages, the postoperative outcomes for stage 4B were noticeably worse than those for stage 4A. The limited visual outcomes in stage 4B patients highlight the need for earlier surgical intervention in stage 4A. Moreover, in the analysis focused on the 4A group, being a phakic eye was identified as a predictor of good BCVA at both ages 5 and 8, independent of the dominant eye. This indicates that within the 4A group, it is preferable to perform surgery early, before the fibrovascular membranes become extensive and complicate vitrectomy without lensectomy.

There have been no prior reports documenting the longitudinal changes in BCVA and refractive changes after ROP surgery. In this study, among the 25 eyes in the poor BCVA group at age 5, 9 eyes (36.0%) achieved good BCVA by age 8. One hypothesis for this improvement is the delayed cognitive development often observed in premature infants. Alred et al. evaluated children with stage 3 or higher ROP and found they were more likely than their peers to have Bayley Scales of Mental and Psychomotor Development Index (PDI) scores 2–3 standard deviations below the expected mean [21]. Additionally, Fieß et al. suggested that low PDI scores at 2 years of corrected age were associated with reduced visual acuity in children aged 4–10, indicating an interaction between neurologic outcomes and visual acuity in former preterm infants [22]. Due to such developmental delays, some patients may not have reached their full visual potential by age 5. Our analysis of the poor BCVA group at age 5 indicated that the dominant eye was associated with improved BCVA. Therefore, it is crucial to continue visual rehabilitation even for those with poor BCVA at age 5, especially if the eye is the dominant one.

To the best of our knowledge, no studies have investigated long-term refractive changes in eyes following vitrectomy for ROP. Conversely, Wiecek et al. reported that myopic progression lasted for over 8.5 years in ROP patients treated with laser photocoagulation [23]. They noted that myopic progression continued beyond age 8 in laser-treated eyes. Similarly, this study found a myopic shift between the ages of 5 and 8 following vitrectomy for ROP. Notably, cases in the poor BCVA group at age 5 were significantly more likely to experience a myopic shift compared to those in the good BCVA group at that age. This indicates that, in addition to previously identified factors associated with myopia, such as axial length and anterior chamber depth [24], blurred vision resulting from low visual acuity may also contribute to myopia progression. Future studies should include milder cases of ROP, such as those treated with only laser or anti-VEGF therapy.

This study had several limitations. First, refractive errors were assessed solely using subjective methods. However, cycloplegic refraction or more objective measurement techniques may provide greater accuracy. In clinical practice, it is challenging to perform objective refractive assessments for all ROP patients. Therefore, in this retrospective study, we adopted subjective refractive values to evaluate a larger number of patients. Second, some cases referred to our hospitals for surgical treatment from remote areas were excluded due to short follow-up periods, which may have introduced bias in the reported outcomes. Additionally, the exclusion of patients with intellectual disabilities could have resulted in selection bias. Data on ocular parameters such as axial length, corneal radius and lens thickness were not available. The neurodevelopmental score was also not investigated, despite the variability in development among ROP patients. However, we believe that the relatively large number of patients with long-term follow-up examined in this study offers valuable insights into visual development after vitrectomy for stage 4 ROP by a single surgeon.

This study demonstrated the possibility of ongoing visual improvement after age 5 in patients who underwent vitrectomy for stage 4A ROP. Because myopic shifts occur during this period, careful management with appropriate refractive correction is essential, even for those with poor BCVA at age 5 years, particularly when the eye is the dominant one.

## Supplementary Information

Below is the link to the electronic supplementary material.

Fig. 4 Supplementary File 1: Figure S1 Impact of lens status and dominant eye on BCVA at ages 5 and 8 for stage 4A ROP BCVA, best-corrected visual acuity (logMAR acuity); BCVA diff (5–8), difference of BCVA between ages 5 and 8; DE, dominant eye at age 5

High resolution image (TIF 78 KB)

Fig. 5 Supplementary File 2: Figure S2 Relationship between GA and BCVA and SE in phakic eyes at ages 5 and 8 for stage 4A ROP BCVA, best-corrected visual acuity (logMAR acuity); BCVA diff (5–8), difference in BCVA between ages 5 and 8; GA, gestational age (day); rS, Spearman’s rank correlation coefficient; SE, spherical equivalent (diopter)

High resolution image (TIF 122 KB)

## Data Availability

Data are available upon reasonable request.
