# Supplementary figures and images for: Long-term visual function and refractive changes after vitrectomy for stage 4 retinopathy of prematurity
Source: Graefes Arch Clin Exp Ophthalmol. 2025 Mar 22;263(7):2041–8. doi: 10.1007/s00417-025-06801-0 (PMC12373698; doi:10.1007/s00417-025-06801-0)

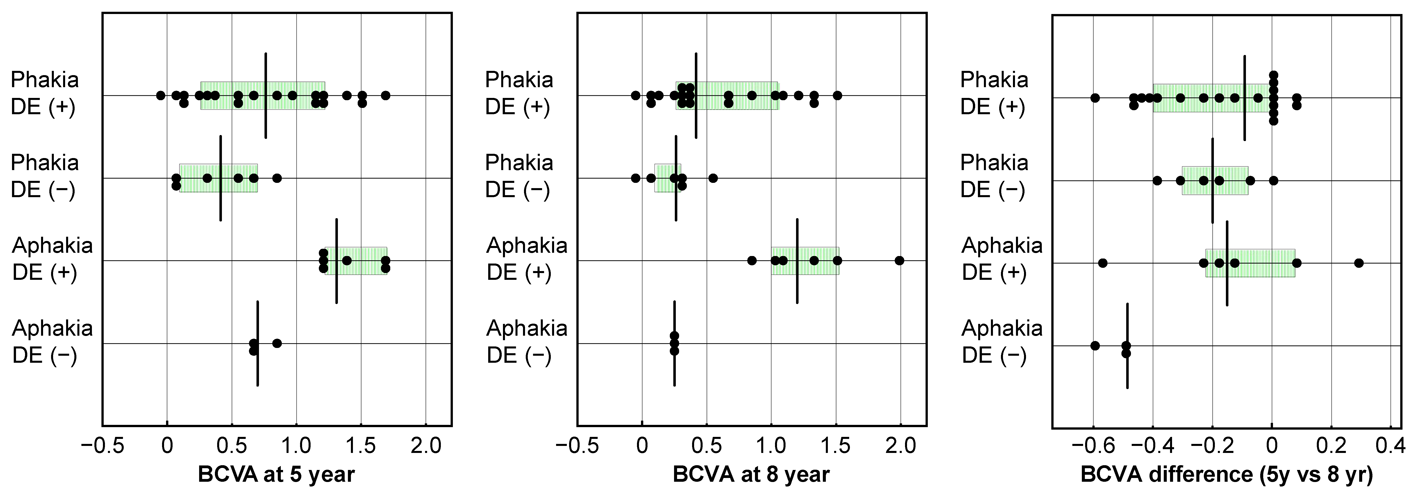

Supplement: Supplementary file 1 — Figure S1 Impact of lens status and dominant eye on BCVA at ages 5 and 8 for stage 4A ROP BCVA, best-corrected visual acuity (logMAR acuity); BCVA diff (5–8), difference of BCVA between ages 5 and 8; DE, dominant eye at age 5 [file 417_2025_6801_Fig4_ESM.png]

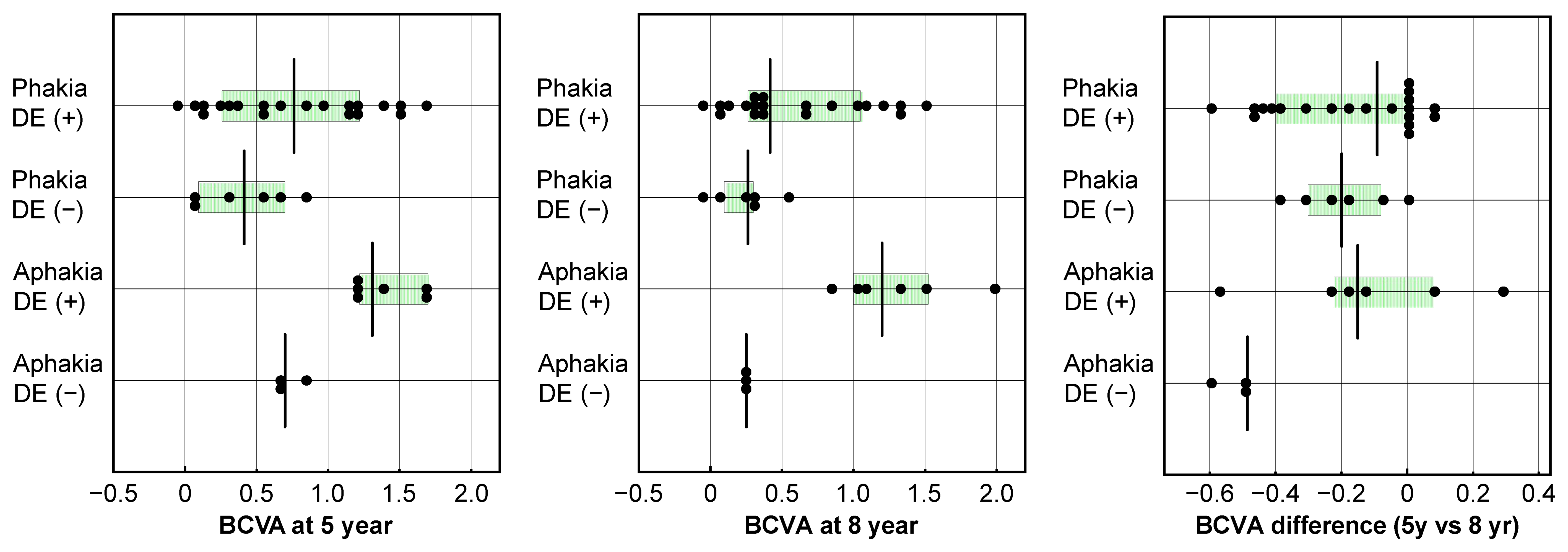

Supplement: Supplementary file 2 — High resolution image (TIF 78 KB) [file 417_2025_6801_MOESM1_ESM.tif]

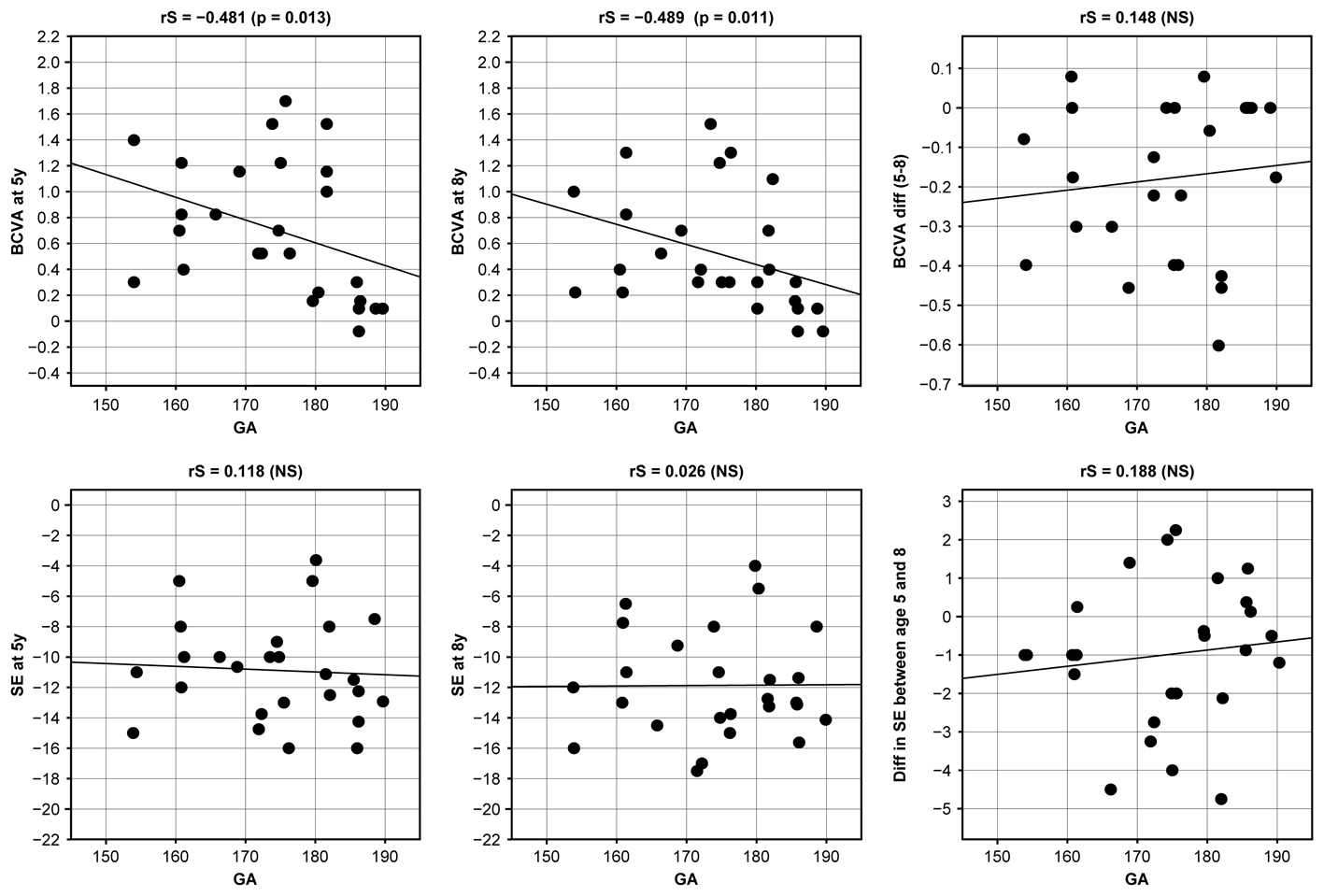

Supplement: Supplementary file 3 — Supplementary File 2: Figure S2 Relationship between GA and BCVA and SE in phakic eyes at ages 5 and 8 for stage 4A ROP BCVA, best-corrected visual acuity (logMAR acuity); BCVA diff (5–8), difference in BCVA between ages 5 and 8; GA, gestational age (day); rS, Spearman’s rank correlation coefficient; SE, spherical equivalent (diopter) [file 417_2025_6801_Fig5_ESM.png]

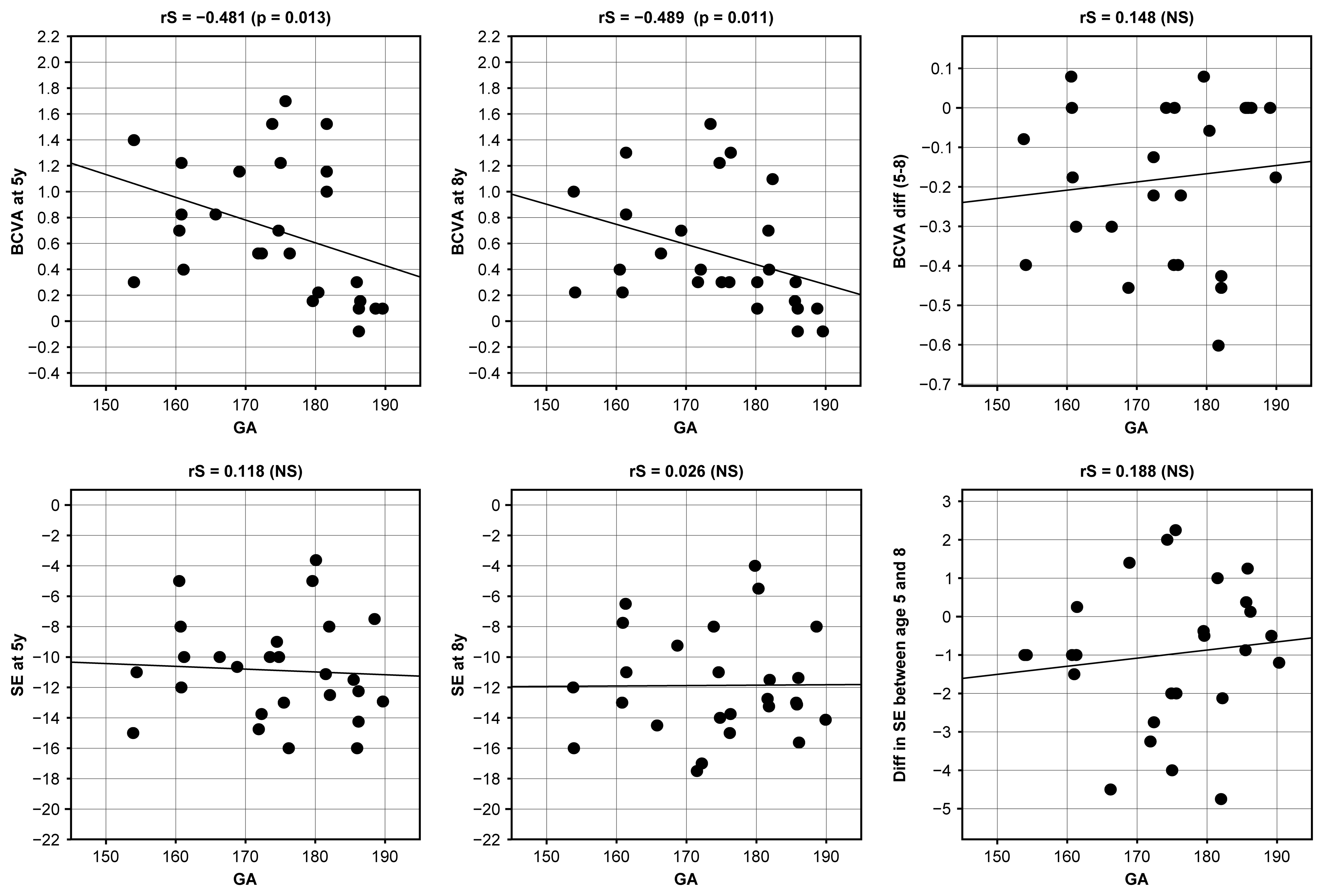

Supplement: Supplementary file 4 — High resolution image (TIF 122 KB) [file 417_2025_6801_MOESM2_ESM.tif]
